# Supplementary material for: Real-Time Analysis of Oxygen Gradient in Oocyte Respiration Using a High-Density Microelectrode Array
Source: Biosensors (Basel). 2021 Jul 29;11(8):256. doi: 10.3390/bios11080256 (PMC8393405; doi:10.3390/bios11080256)
Supplement: Supplementary file 1 [file biosensors-11-00256-s001.zip › SupplementaryMaterials.pdf]

Supplementary Materials

Real-Time Analysis of Oxygen Gradient in Oocyte Respiration Using a High-Density Microelectrode Array

William Tedjo [1], Yusra Obeidat [2], Giovana Catandi [3], Elaine Carnevale [3], Tom Chen [1,4,*]

S1. Biosensor System General Specifications

CMOS Microchip	Value	Units
WE count	16,064	
WE coverage	17.5×17.5	μm
WE perimeter	160	μm
WE surface area	427.5	μm^2
WE pitch (X and Y)	27.5	μm
MEA coverage	3.6×3.6	mm
Read channel count	16,064	
Read channel gain	20	MΩ
Input current range	±80	nA
Temperature	38.0	°C
Temperature tolerance	±0.5	°C
Power dissipation	950	mW
**Potentiostat**	**Value**	**Units**
Max RE voltage range	±2.74	V
Max AE voltage range	±2.74	V
Max CV sweep rate	5	kV/s
Max voltage error	<1	mV
**System**	**Value**	**Units**
Frame capture rate	4	frames/s
Limit of Detection of pO_2	18.3	μM
	0.58	mg/L
	13.8	mmHg
	150.4	pA
MEA microfluidic chamber volume	3.9	μL
Supporting PCBs Power dissipation	180	mW
Overall Power dissipation	1,130	mW

Table S1. Biosensor system general specifications.

S2. Graphical User Interface (GUI)

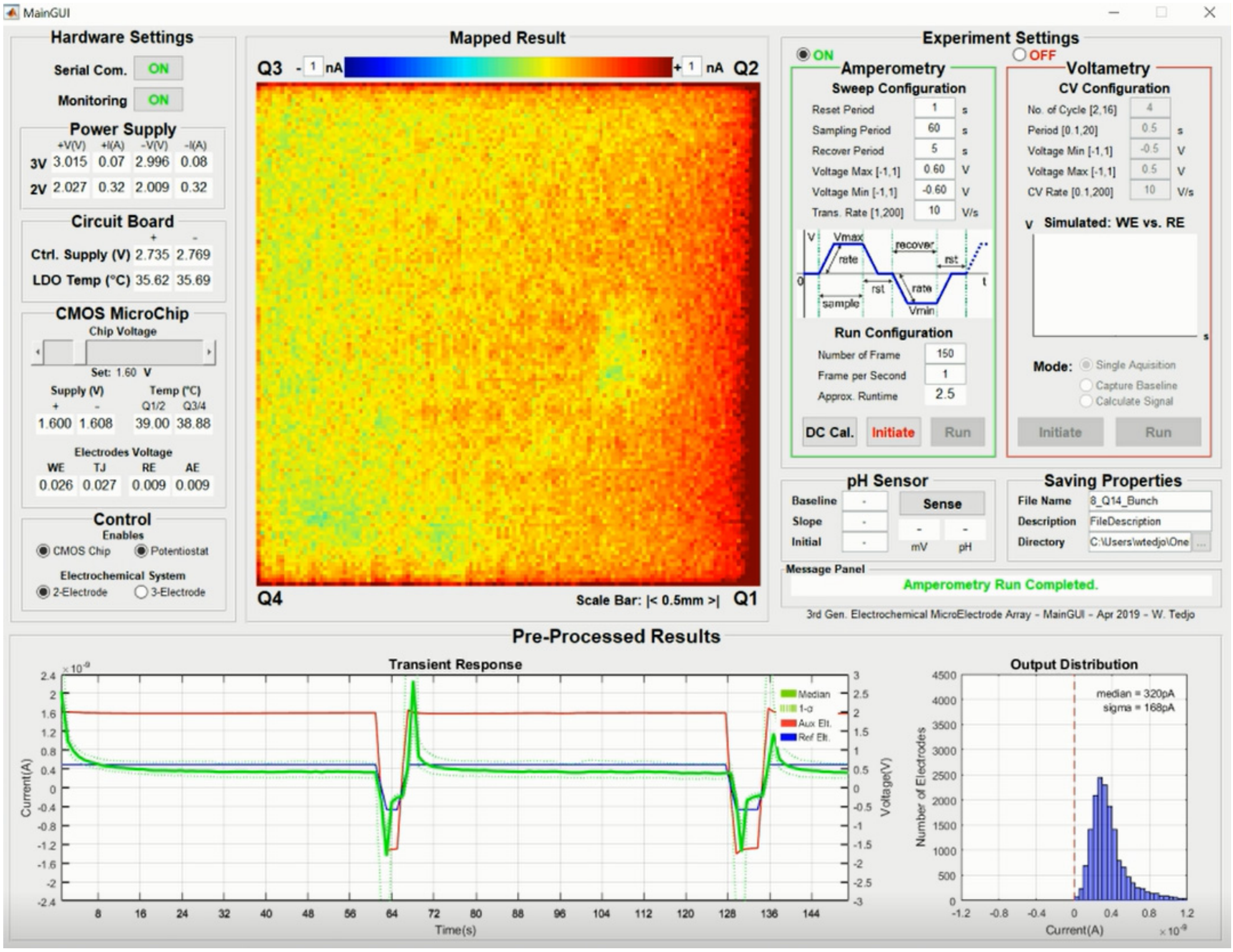

Figure S2(A). A screenshot of the custom GUI during an actual oxygen reading of bovine cumulus-oocytes-complexes (COCs). The Main GUI provides the user with various kind of control, monitoring, and selections of electrochemical analysis methods, amperometry or voltammetry. In this specific experiment, the GUI shows real-time heatmap representation, transient response, and instantaneous histogram output at 1Hz update rate for a duration of 2.5 minutes. The amperometry mode were set to have 60s sampling (V_{RE} = -0.6V), 1s reset (V_{RE} = 0V), and 5s recover (V_{RE} = 0.6V).

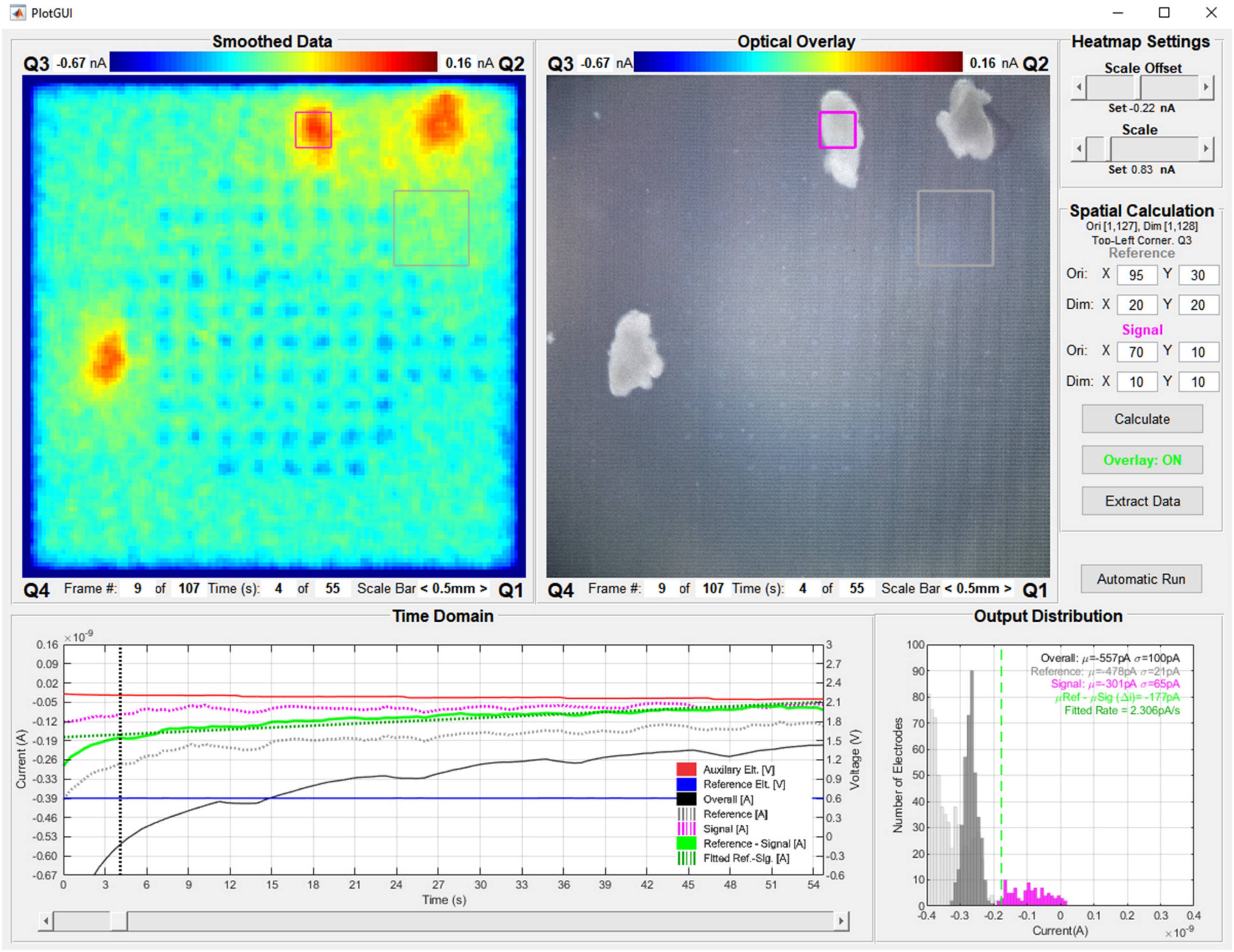

Figure S2(B). A screenshot of the custom GUI during the 2D data analysis of COCs oxygen consumption. The Plot GUI provides the user with controls over selecting the locations and sizes of the electrode group for signal (magenta box) and reference (grey box) analyses at different time points. The value of signal and reference are compared and plotted over time and depicted as histograms at the selected time point. The Plot GUI accelerates the process of data analysis by allowing the user to conveniently select the signal and reference regions and provide real-time statistical information accordingly.

S3. Calculation of MEA Oxygen Reduction

Known values:

- Microfluidic channel cross section volume: 3.9uL
- Concentration of dissolved oxygen (O2) at local atmospheric pressure: 165uM = 165umol/L
- 1 mol = 6.022×10^{23} molecules
- Number of available O2 molecules in the microfluidic chamber:
  165 umole/L x 3.9 uL = 0.6435×10^{-9} mol
  0.6435×10^{-9} mol = (0.6435×10^{-9}) * $(6.022 \times 10^{23}$ molecules) = 3.875×10^{14} molecules
- Total charge consumed (in Coulomb) as a result of O_2 reduction, assuming each O_2 molecule consume four electrons. Oxygen reduction: $O_2 + 4H^+ + 4e^- \rightarrow 2H_2O$
- 1 e- = 1.602×10^{-19} C
- 1C = 6.2415×10^{18} negatively charge electron (e-)

Therefore, available electron in the chamber:

- 3.875×10^{14} O_2 molecules * 4e- = 1.550×10^{15} e-
- 1.550×10^{15} / 6.2415×10^{18} = 248.35 uC for the whole array

Charge being consumed by a single WE:

248.35 uC/16064 = 15.46 nC

Case 1 (duty cycle: 72.5%):

integral of -0.7/10^9*exp(-0.06*t) for t = 0 to 75

$$\int_0^{75} -\frac{0.7\exp(-0.06\,t)}{1\times 10^9}\,dt = -1.15371\times 10^{-8}$$

Total charge per WE = 11.54 nC * 72.5% = 8.37 nC

Case 2 (duty cycle: 25%):

integral of -0.7/10^9*exp(-0.023*t) for t = 0 to 135

$$\int_0^{135} -\frac{0.7\exp(-0.023\,t)}{1\times 10^9}\,dt = -2.90706\times 10^{-8}$$

Total charge per WE = 29.07 nC * 25% = 7.27 nC

Case 3 (duty cycle: 12.5%):

integral of -0.7/10^9*exp(-0.012*t) for t = 0 to 275

$$\int_0^{275} -\frac{0.7\exp(-0.012\,t)}{1\times 10^9}\,dt = -5.61818\times 10^{-8}$$

Total charge per WE = 56.18 nC * 12.5% = 7.02 nC

S4. Heatmap Data Processing

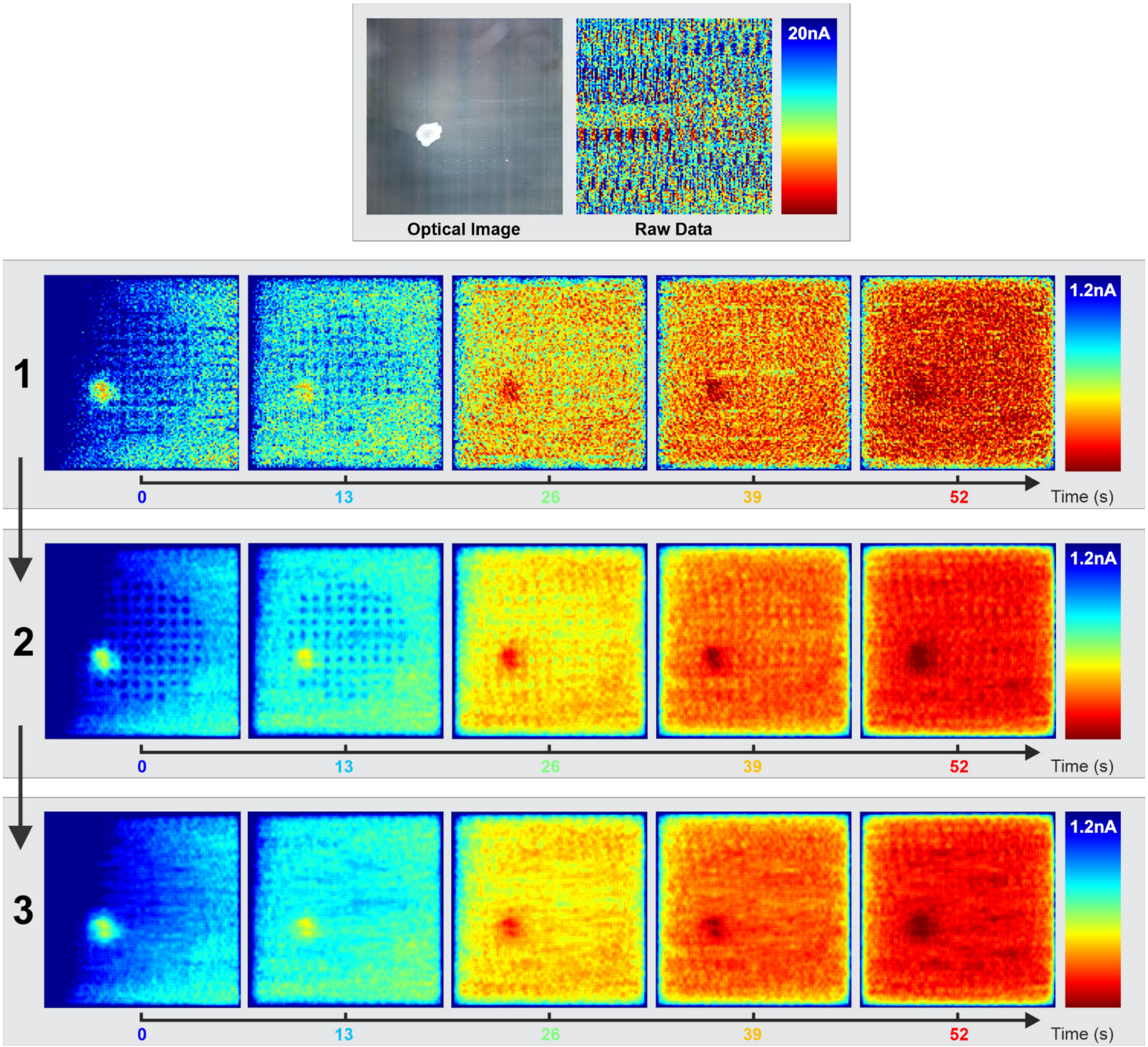

Figure S4. Progression of data processing for generating heatmaps.

Step 1 – Heatmap after baseline offset calibration.

The baseline offset is due to the electrical circuit variations, which are constant signature values from each 16 thousand read channels with no noticeable drift over tens on minutes and tens of ºC temperature variations. A COC and Utility Electrodes (UEs) patterns become visible in this step.

During an imaging experiment, the baseline calibration is manually performed at the beginning to remove the residual DC offset error due to random leakage at each WEs as described previously. The DC calibration should be performed when the system is at a steady-state and stable environmental and electrical condition, and within every imaging experiment run. The process of the spatial baseline calibration follows this algorithm step:

First, Electrode Array (A) configuration in x-y coordinates is defined by:

$$\boldsymbol{A}_{xy} = \begin{bmatrix} a_{11} & \cdots & a_{1y} \\ \vdots & \ddots & \vdots \\ a_{x1} & \cdots & a_{xy} \end{bmatrix}$$

$$1 \le x \le 128\,, \qquad 1 \le y \le 128$$

Electrode array *Data Raw (DR)* over the *time (t)* is defined by:

$$\boldsymbol{DR_{xyt}} = \begin{bmatrix} dr_{11t} & \cdots & dr_{1yt} \\ \vdots & \ddots & \vdots \\ dr_{x1t} & \cdots & dr_{xyt} \end{bmatrix}$$

$$1 \leq t \leq \#\ of\ frame\ in\ an\ experiment$$

Baseline Calibration (BC) data is captured at the beginning of experiment (dry electrode array) over specified number of frames (f), and defined as:

$$\begin{bmatrix} bc_{11} & \cdots & bc_{1y} \\ \vdots & \ddots & \vdots \\ bc_{x1} & \cdots & bc_{xy} \end{bmatrix} = \boldsymbol{BC_{xy}} = \frac{1}{nbase} \sum_{f=1}^{nbase} \begin{bmatrix} bc_{11f} & \cdots & bc_{1yf} \\ \vdots & \ddots & \vdots \\ bc_{x1f} & \cdots & bc_{xyf} \end{bmatrix}$$

$$f = \#\ of\ frame\ for\ baseline\ calculation\ (nbase)$$

Calibration of baseline; generates *Data Baselined (DB)* output:

$$\begin{bmatrix} bc_{11t} & \cdots & bc_{1yt} \\ \vdots & \ddots & \vdots \\ bc_{x1t} & \cdots & bc_{xyt} \end{bmatrix} = \boldsymbol{DB_{xyt}} = \sum_{t=1}^{stop} (DR_{xyt} - BC_{xy})$$

Therefore,

$$\boldsymbol{DB_{xyt}} = \sum_{t=1}^{stop} \left(\begin{bmatrix} dr_{11t} & \cdots & dr_{1yt} \\ \vdots & \ddots & \vdots \\ dr_{x1t} & \cdots & dr_{xyt} \end{bmatrix} - \frac{1}{nbase} \sum_{f=1}^{nbase} \begin{bmatrix} bc_{11f} & \cdots & bc_{1yf} \\ \vdots & \ddots & \vdots \\ bc_{x1f} & \cdots & bc_{xyf} \end{bmatrix} \right)$$

$$1 \leq x \leq 128\,, \quad 1 \leq y \leq 128, \quad t = \#\ of\ frame\ in\ an\ experiment\ until\ (stop)$$

This step concludes the baseline calibration process. DB is shown in real-time in the GUI, while BC, is saved together with the other data and information for further data analysis processes.

Step 2 – Heatmap after temporal and spatial smoothing.

The spatial smoothing is in a form of simple vertical and horizontal moving averaging with a 3-pixel window. The moving averaging can be set to a higher odd number of pixel windows (e.g. 5, 7, etc.) for a smoother spatial gradient effect, however, with a cost of losing spatial resolution.

The temporal smoothing follows a moving averaging of at least 11 frames (over a duration of few seconds) of a single WE. The smoothing is individually applied to each WE over the duration of the experiment data.

Step 3 – Heatmap after UEs artifact removal.

The pixels at and surrounding UEs are detected and replaced by the pixels' values in nearby proximity. Considering the diagram from Figure 1D and E, the UE is in a fixed 2x2 size of the WE uniformly located in a circular pattern of 80 UEs. An extra margin of two pixels surrounding each UE is added to account for the diffusion effect of lack of oxygen reduction surrounding each UEs. The two pixels margin was experimentally selected to properly filtered out the UE artifact without losing too much important spatial data. Therefore, there's 4x4 pixel replacement for each UE artifact removal. At last, the 4x4 data is generated by extrapolating two nearby pixels value outside each 4x4 pixel.

The order of Step 2 and Step 3 are preferred in this specific application; however, one may also consider UEs artifact removal before temporal and spatial smoothing to optimize the imaging results.

S5. Heatmap Video – Healthy COC with Flow

File Name: HealthyCOCwithFlow.avi

The heatmap video shows a healthy COC being flushed by fully saturated pO_2. The response shown in the video corresponds to the discussion in section "Comparison of Healthy and Dead COC" and Figure 6A. This video is without smoothing post-processing step 3 defined in Supplementary Information 4.

Timeframe of the pO_2 gradient measurement:

Time (s)	Description
0	A healthy COC was placed close to the inlets on the left side of the MEA. Potentiostat in amperometry mode is activated.
5	First sign of COC oxygen reduction. UE area shows lack of oxygen reduction.
6 - 25	Continuous reduction of the whole MEA, COC oxygen reduction is still apparent.
36 - 40	COC oxygen reduction becomes less apparent as the MEA homogenously reduced the oxygen.
41 - 55	A sudden flow of 40 μL/min fully saturated pO_2 media was introduced. The cell was pushed to the right (~250 μm) by the forced flow.
56	Flow was stopped.
60	Any sign of leftover forced flow and turbulence disappeared.
61 – 104	Repeats the process of t = 0 to 40 s.

S6. Heatmap Video – Multiple Cells Measurement

File Name: MultipleCellsMeasurement.avi

The heatmap video shows multiple COCs under oxygen measurement discussed in section "Oxygen Flux and Consumption Rate Analysis" and Figure 7. This video is without smoothing post-processing step 3 defined in Supplementary Information 4.
