# Supplementary figures and images for: Real-Time Analysis of Oxygen Gradient in Oocyte Respiration Using a High-Density Microelectrode Array
Source: Biosensors (Basel). 2021 Jul 29;11(8):256. doi: 10.3390/bios11080256 (PMC8393405; doi:10.3390/bios11080256)

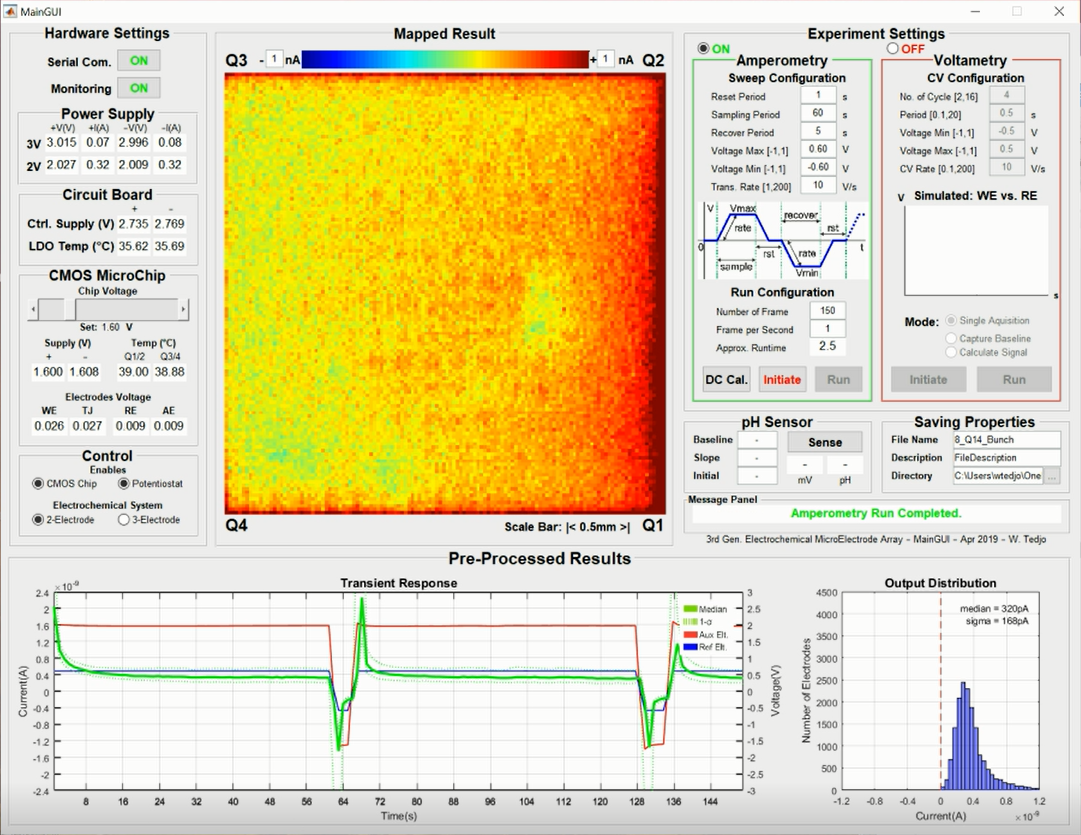

Supplement: Supplementary file 1 [file biosensors-11-00256-s001.zip › FigS2A.png]

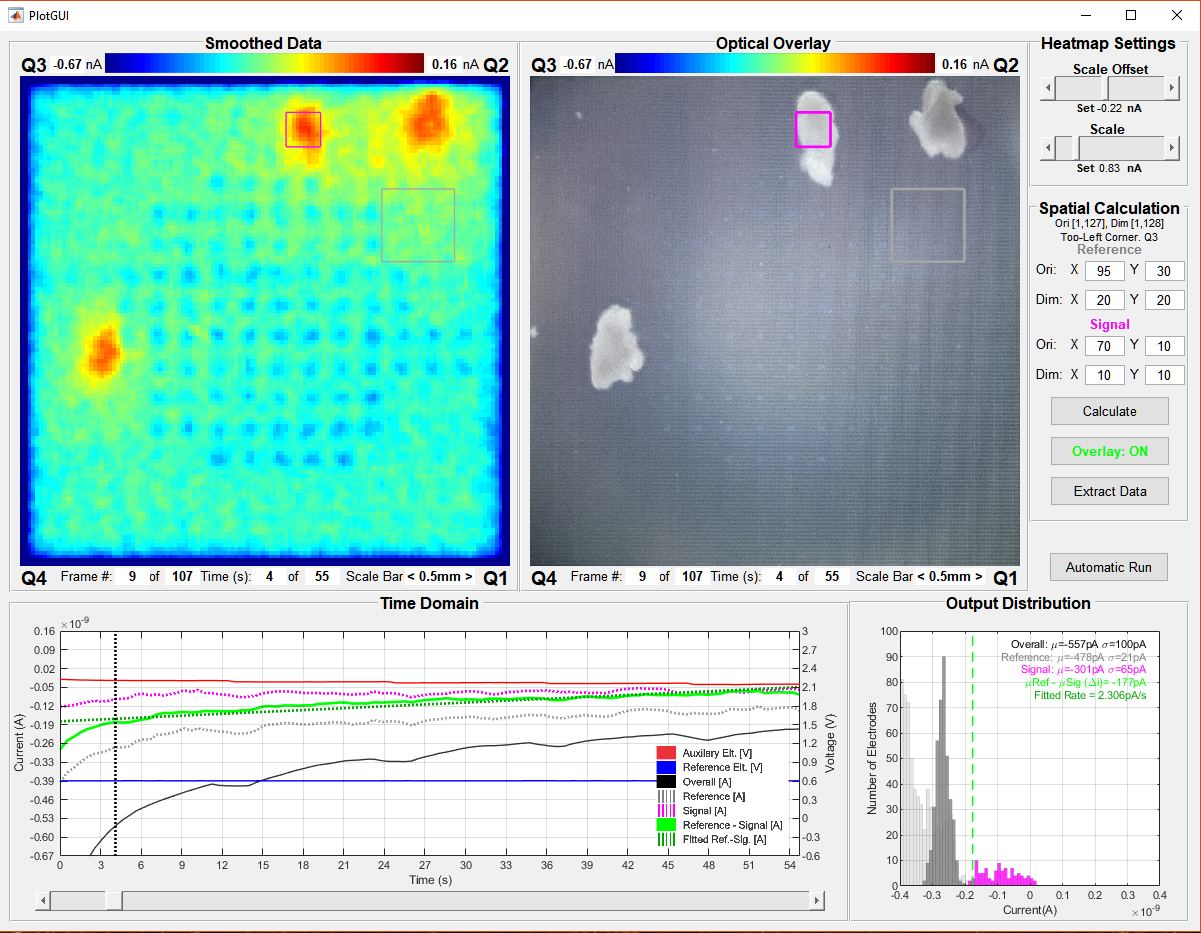

Supplement: Supplementary file 1 [file biosensors-11-00256-s001.zip › FigS2B.png]

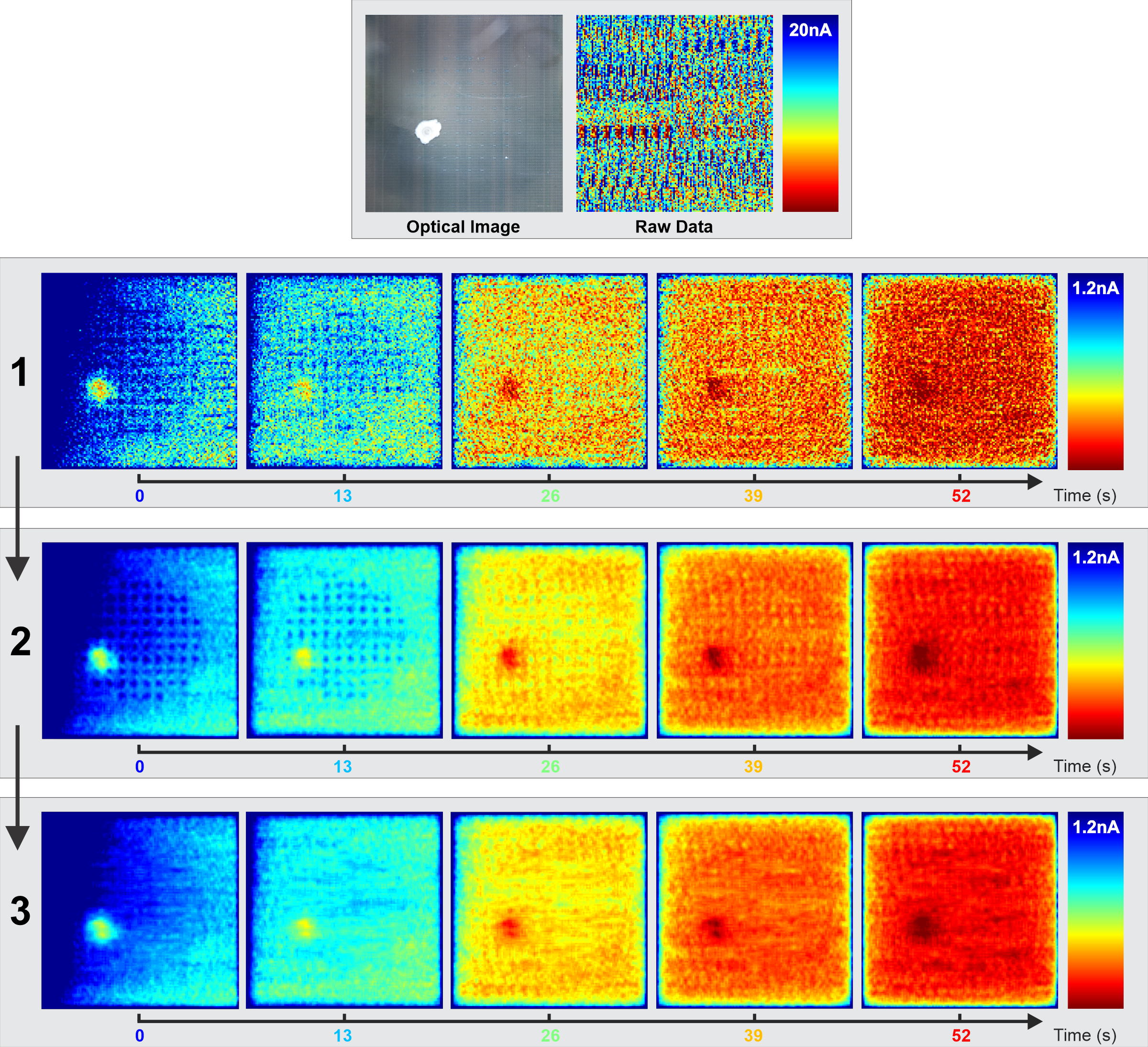

Supplement: Supplementary file 1 [file biosensors-11-00256-s001.zip › FigS4.png]
